# Association of alcohol consumption and rotator cuff retear: a case control matched cohort study

**DOI:** 10.1007/s00402-025-05771-6

**Published:** 2025-02-05

**Authors:** Suchung Kim, Kerstina Menzel, Lucca Lacheta, Philipp Moroder, Jan Dekena, Doruk Akgün, Kathi Thiele, Katrin Karpinski

**Affiliations:** 1 Orthopädie Berlin, Privatpraxis - OrthoEins, Dr. Topar, Berlin, Germany; 2 Charité - University Medicine Berlin, Berlin, Germany; 3 Technical University of Munich, Munich, Germany; 4 Schulthess-Klinik, Zurich, Switzerland

**Keywords:** Arthroscopic rotator cuff repair, Rotator cuff retear, Alcohol consumption

## Abstract

**Background:**

Failure of healing or retear after surgical repair of the rotator cuff tendons are still a problem and can cause ongoing shoulder pain and dysfunction. Compromised microcirculation as seen in regular alcohol consumption may lead to poor healing.

**Purpose:**

To compare the clinical outcomes and tendon integrity of patients after rotator cuff repair with and without regular alcohol intake.

**Study design:**

Case control matched cohort study; Level of evidence, 3.

**Methods:**

Patients who underwent arthroscopic rotator cuff repair (ARCR), had regular alcohol intake (according to world health organization (WHO) definition of harmful alcohol consumption) and were at least 2 years postoperative were included, and matched according to age, sex, involved tendon, and tear size with patients who underwent ARCR without regular alcohol intake. Patient-reported outcome (PRO) scores were collected at final follow-up including the Constant Murley Score (CMS), Western Ontario Rotator Cuff Score (WORC), Simple Shoulder Test (SST), and visual analog scale (VAS). Tendon integrity (maintained continuity: yes/no = full thickness) was assessed by ultrasound examination at final follow-up. Complications and revision surgeries are reported.

**Results:**

Twenty-two patients (versus twenty-two matched—controls) were available for follow-up. There were two female (9%) and twenty male (91%) patients with a mean age of 66.6 years (standard deviation, 36–85 years). The mean follow-up was 4 years (standard deviation, 2–5 years) in the alcohol group and 5 years (standard deviation, 2–10 years) in the non-alcohol group. No differences in mean PRO scores between alcohol and non-alcohol groups were seen except VAS (0.5 (standard deviation, 0–5) vs. 1.6 (standard deviation, 0–8), (*P* = 0.049*) respectively). Intact tendon insertion was seen in 77% (17/22) for the alcohol group and 100% (22/22) for non-alcohol group, (*P* = 0.021*). One patient underwent revision surgery (5%) in the alcohol group due to a retear, no further peri- or postoperative complications were noticed.

**Conclusion:**

Patients with torn rotator cuff tendons benefited similarly from ARCR independently of their alcohol use concerning clinical presentation. However, significantly higher retear rates were recorded in the alcohol group.

## Introduction

Although arthroscopic techniques have improved in recent years with high biomechanical stability high rates of retears have been recorded leading to worse outcomes compared to patients with an intact cuff [1].Therefore, the biology and the ability of tendon healing seems to be the weak link following rotator cuff repair. Massive rotator cuff tears account to 40% of all rotator cuff tears [2]. Although the number of rotator cuff repairs has increased in recent years [3–5] due to improved technologies and expanded indications, the surgical technique is demanding, and re-tear rates are high. Clinically, the best results following treatment of massive rotator cuff tears is arthroscopically repair of the force couples, compared to other techniques. Potential to tendon repair depends on different patient related factors and tendon condition. Patient´s age, type and size of tear, status of the bony part of glenohumeral joint, as well as preexisting muscle fatty infiltration and atrophy contribute to reparability [2, 6]. One of the largest database based on a cohort study by [7] including 5000 patients after arthroscopic rotator cuff repair have assessed the relative contributions of comorbidities and several risk factors for rotator cuff disease. Despite different factors such as current or previous smoking history, body mass index of greater than 30, any alcohol intake, medial epicondylitis, de Quervain syndrome, cubital tunnel syndrome, and rheumatoid arthritis were shown to be associated with rotator cuff disease, alcohol intake seemed not to be a crucial risk factor for rotator cuff disease. However, this is in contrast to Passaretti et al. [8] who have shown that long-term alcohol intake may be a significant risk factor for the occurrence and severity of rotator cuff tear in both sexes. Negative effects of chronic alcohol abuse on biological healing particularly its disruptive impact during inflammatory response have been largely investigated in a previous study by [9]. Bone metabolism plays a crucial role in the risk management of rotator cuff tears. Previous studies have shown that patients with compromised bone quality, such as patients with osteoporosis, have a higher risk of diminished tendon-to-bone healing [10] and rotator cuff re-tears [11–13], which should be taken into account when counseling patients. Little is known about the effect of chronic alcohol consumption on the outcome of patients after arthroscopic rotator cuff repair (ARCR). Therefore, the purpose of this study was to compare the clinical outcomes and tendon integrity in patients who underwent ARCR with regular alcohol intake to those who have no regular alcohol consumption. The hypothesis was that chronic alcohol intake would show negative effect on both clinical outcomes as well as on tendon healing.

## Methods

In this retrospective, single-center study approval was granted by the institutional review board (anonymized).

### Patient selection

A retrospective medical chart review was performed. Included were all patients before 2018 who underwent ARCR, had regular alcohol intake at time of surgery and postoperative, and had at least 2 years of follow-up. Regular alcohol intake was defined as more than 24 g alcohol per day for men and more than 12 g alcohol per day for women according to the WHO guideline for harmful alcohol consumption. Patients were matched 1:1 with patients who underwent ARCR without regular alcohol or drug intake by age, sex, involved tendon, and tear size. Patients who had earlier surgery of the affected shoulder, presented with signs of osteoarthritis in radiographs at times of surgical intervention greater than Samilson/Pietro grade 1 as well as comorbidities (e.g. diabetes or hypothyroidism) were excluded in both groups. All patients were asked once by telephone to take part in this study. Written consent was provided at the scheduled follow-up appointment for a clinical and radiological examination.

### Clinical evaluation

At final follow-up, all patients were evaluated by a single investigator who was not involved in the surgical procedures. Patient-reported outcome (PRO) scores were collected including the Constant–Murley Score (CMS) [14], Western Ontario Rotator Cuff Score (WORC) [15], Simple Shoulder Test (SST) [16], and visual analog scale (VAS). A handheld isobex isometric dynamometer (Isobex^®^ Cursor AG, Bern, Switzerland) was used to evaluate isometric muscle strength. Regularity and quantity of alcohol intake was enquired.

### Assessment of tendon integrity

Tendon integrity (maintained continuity: yes/no) was assessed by ultrasound examination at final follow-up [17, 18]. In ultrasound examination of the shoulder joint standardized planes were employed transverse and longitudinal in the dorsal, superior/lateral and ventral region. These planes were recorded by using a 7.5 MHz linear transducer in correspondence to the guidelines provided by the German Society of Ultrasonography (DEGUM). The ultrasound examinations were performed by the examinator and independently confirmed by the senior physician specialized in advanced shoulder surgery. The tendon appearance was grouped as follows: Normal homogenous appearance throughout, and where it inserts onto the lesser or greater tuberosity or lucent patch visualized in the tendon either on the articular or bursal surface, but continuous tendon fibers inserting into the lesser or greater tuberosity were classified as an intact rotator cuff repair. A lucent patch is visualized running through the full thickness of the tendon with abnormal tendon enthesis, and the surface of the tuberosities filled with heterogenous substance at the space left by the tendon, was classified as re-torn rotator cuff (no tendon continuity).

### Statistical analysis

Statistical analyses were performed with SPSS (IBM SPSS Statistics 24). A p-value < 0.05 was considered significant. Descriptive statistics were calculated, including means, standard deviation, and minimum and maximum values of continuous variables. The unpaired T test or the Mann–Whitney test was used to compare clinical results between treatment groups. Categorical variables were evaluated using the chi-square test or Fisher exact test.

## Results

Thirty-eight patients were found who reported regular alcohol intake according to the aforementioned definition. Twenty-two patients (versus twenty-two matched – controls) were available for follow-up at mean of 4 years (standard deviation, 2–5) in the alcohol group and at mean 5 years (standard deviation 2–10) in the non-alcohol group. Patient variables are presented in Table 1 and quantity of alcohol intake for the alcohol group are presented in Table 2. One patient underwent revision surgery (5%) in the alcohol group due to a retear, no further peri- or postoperative complications were noticed.

**Table 1:**
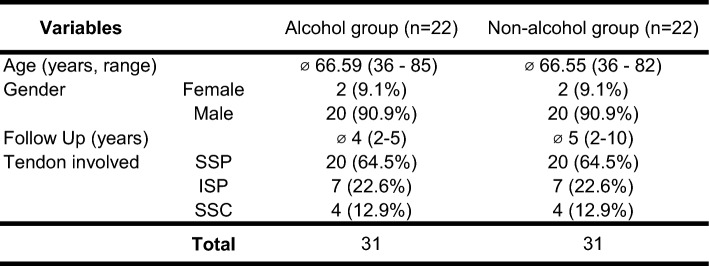
Patient variables

Patient varibales: SSP,  Supraspinatus tendon; ISP,  Infraspinatus tendon; SSC,  Subscapularis tendon

**Table 2:**
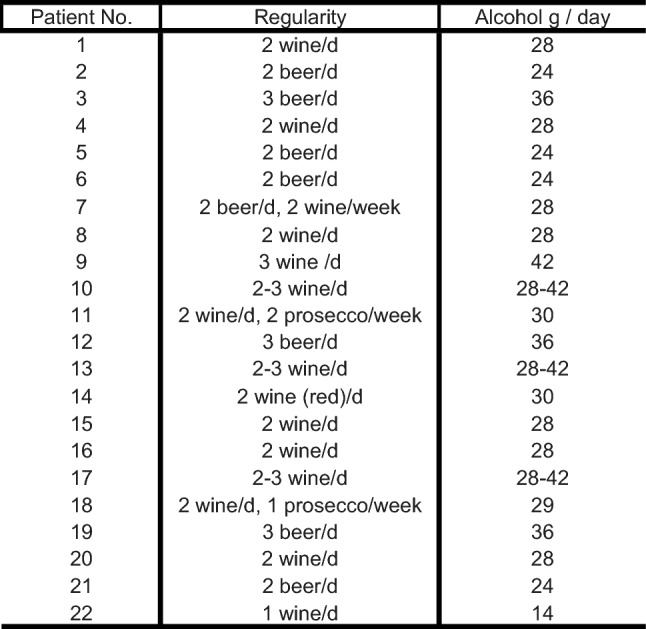
Alchol consumption

No., patient number; d, day; g, gramm

### Clinical results

No differences in mean PRO scores were observed between alcohol and non-alcohol groups, except VAS [0.5 (standard deviation, 0–5) vs. 1.6 (standard deviation, 0–8) (*p* = 0.049*)] (Table 3), respectively, were seen at final evaluation Follow-up ultrasound assessment demonstrated a full thickness retear in 33% of the cases (5\22) in alcohol group and no retear in non-alcohol group (*p* = 0.021*).

**Table 3:**
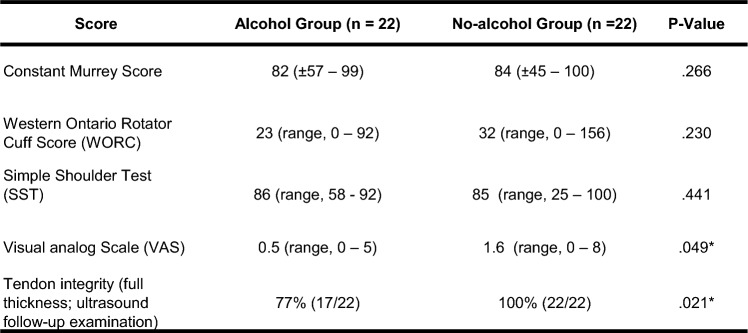
Functional outcome and tendon integrity

^a^values are expressed as mean ± SD; *P < 0.05

## Discussion

The results of our study show comparable satisfactory results in PROs after ARCR irrespective of alcohol use, however a significantly increased retear rate in patients with regular alcohol intake. Evidence of the effect of regular alcohol consumption on the risk for rotator cuff tears lack and comparability across the studies is limited. One study by Rechardt et al. [19] found no association between alcohol consumption and rotator cuff tendinopathies. A case–control study by Passaretti et al. [8] with 249 consecutive patients reported from patients with history of long-term alcohol intake with significantly higher risk for rotator cuff tear compared to non–to moderate alcohol consumers (OR = 1.9, 95% CI 0.94–4.1). A study by Hapa et al. [20] investigated the effect of ethanol on the achilles tendon in rat models after regularly administering ethanol. Results provided increased healing disorders with tendon load failures after 3 weeks. underlining the toxic effect of alcohol on fibroblast proliferation and collagen synthesis through abnormal tenocyte morphology. On the very basis, alcohol consumption is known to negatively influence the capillary microcirculation and tissue perfusion [21–26] which might have a greater impact on tendons due to its hypovascularized origin. This might lead to an increased number of retears in the group of regular alcohol intake in the present study, even when PROs were not seen different between the groups which in turn might be explained by short follow up periods and may alter in longer follow up times.

Compliance is crucial in disease management. Based on decades of research in the United States half of patients fail complying with physicians´ prescribed treatment and medications or lacks consistency in following recommendations [27, 28]. While different causative factors have been investigated in the past, chronic alcohol intake and excessive misuse were found to be strongly related to medical incompliance [29–32].This is of concern, since a successful surgery is obtained, not only by surgery itself but also by patient´s compliance in postsurgical after-care. Otherwise, complications such as healing disorders or retears are at elevated risk for the patient. Consequently, the retear rate in the present study might be also explained by inconsistent following of immobilizing precaution after ARCR. Furthermore, a chronic alcohol intake or excessive misuse is interpreted to reduce pain intensity. A meta-analysis by Thompson et al. [33] considered clinically relevant analgetic effect of alcohol which might explain the comparable good results in PROs in our study. Future studies with longer follow-up periods might provide more reliable PROs. Throughout the reduced pain intensity physical precaution is lowered and actual results in PROs are distorted. Therefore, chronic alcohol consumption should be considered and critically scrutinized in preoperative consultation of patients with acute rotator cuff tears (see Table 3).

There are some limitations in the present study. Major limitation is that evaluating alcohol consumption in medical context patients tend to underreport the total amount of alcohol intake due to stigma typically related to abnormal consumption. Secondly, compliance/noncompliance plays a crucial role in the treatment of patients with chronic alcohol consumption. Therefore, an alternative patient management might help in that patient cohort. For example, more frequent follow examinations incorporating sonographic assessment of reattached tendon structure that may allow differentiation between incompliant patients particularly in early stages and patients with over motivated patients with depicting signs of an overuse in sense of chronic inflammation at later postsurgical stages. Third, the sample size in our study is small compared to earlier studies. This might have an impact on the PROs and rate of retears indicating once more greater sample size with longer follow up periods may alter the mean PROs at later stages.

## Conclusion

Patients with torn rotator cuff tendons benefited similarly from ARCR concerning clinical presentation. However, significantly higher retear rates were recorded in the alcohol group. Further studies with greater sample sizes and longer follow-up are needed to investigate potential functional impairment of patients with regular alcohol consumption. In addition, more frequent follow-up examinations in the early stages after surgery with sonographic assessment may help to objectify overuse in patients in the alcohol group who have less pain.

## Data Availability

No datasets were generated or analysed during the current study.

## Abbreviations

ARCR: Arthroscopic Rotator Cuff Repair
CMS: Constant Murley Score
CI: Confidence Interval
DEGUM: *Deutsche Gesellschaft für Ultraschall in der Medizin* (German Society of Ultrasonography)
IBM: International business machines Corporation
MHz: Megahertz
PRO: Patient-reported outcome
OR: Odds Ratio
SST: Simple shoulder test
WHO: World Health Organization
WORC: Western Ontario Rotator Cuff Score
VAS: Visual analog scale
