# Correction: Association of alcohol consumption and rotator cuff retear: a case control matched cohort study

**DOI:** 10.1007/s00402-025-05885-x

**Published:** 2025-07-03

**Authors:** Kerstina Menzel, Lucca Lacheta, Philipp Moroder, Jan Dekena, Doruk Akgün, Kathi Thiele, Katrin Karpinski, Suchung Kim

**Affiliations:** 1 Orthopädie Berlin, Privatpraxis - OrthoEins, Dr. Topar, Berlin, Germany; 2 Charité - University Medicine Berlin, Berlin, Germany; 3 Technical University of Munich, Munich, Germany; 4 Schulthess-Klinik, Zurich, Switzerland

**Correction: Archives of Orthopaedic and Trauma Surgery (2025) 145:158**

10.1007/s00402-025-05771-6

In the original version of this article, order of authors “Kerstina Menzel, Lucca Lacheta, Philipp Moroder, Jan Dekena, Doruk Akgün, Kathi Thiele, Katrin Karpinski, Suchung Kim” were incorrectly written as “Suchung Kim · Kerstina Menzel · Lucca Lacheta · Philipp Moroder · Jan Dekena · Doruk Akgün · Kathi Thiele · Katrin Karpinski”.

The affiliation details for author Katrin Karpinski were incorrectly given as

^1^Orthopädie Berlin, Privatpraxis - OrthoEins, Dr. Topar, Berlin, Germany

^2^Charité - University Medicine Berlin, Berlin, Germany

^3^Technical University of Munich, Munich, Germany

but should have been

^2^Charité - University Medicine Berlin, Berlin, Germany

